# Chord skill: learning optimized hand postures and bimanual coordination

**DOI:** 10.1007/s00221-023-06629-2

**Published:** 2023-05-14

**Authors:** Willem B. Verwey

**Affiliations:** Department of LDT-Section Code, Faculty of Behavioural, Management and Social Sciences, University of Twente, PO Box 217, 7500 AE Enschede, The Netherlands

**Keywords:** Chording, Information processing, Motor learning, Interference between fingers

## Abstract

This reaction time study tested the hypothesis that in the case of finger movements skilled motor control involves the execution of learned hand postures. After delineating hypothetical control mechanisms and their predictions an experiment is described involving 32 participants who practiced 6 chord responses. These responses involved the simultaneous depression of one, two or three keys with either four right-hand fingers or two fingers of both hands. After practicing each of these responses for 240 trials, the participants performed the practiced and also novel chords with the familiar and with the unfamiliar hand configuration of the other practice group. The results suggest that participants learned hand postures rather than spatial or explicit chord representations. Participants practicing with both hands also developed a bimanual coordination skill. Chord execution was most likely slowed by interference between adjacent fingers. This interference seemed eliminated with practice for some chords but not for others. Hence, the results support the notion that skilled control of finger movements is based on learned hand postures that even after practice may be slowed by interference between adjacent fingers.

## Introduction

A major question in motor research concerns how the brain controls movement. For instance, in the case of pressing a key with a finger there are indications that instead of activating muscles of just that finger, control may involve the selection and execution of a posture of the hand or even of the entire hand-arm system. Similarly, executing one of a repertoire of stored hand postures would allow grasping peculiarly shaped objects and playing musical instruments. Below, several mechanisms are discussed that might be involved in the control of finger movements. Then an experiment is reported aimed at disentangling the contributions of these mechanisms to finger movements in a laboratory chord task.

The chord task in the present study received this name because it mimics the chords executed by musicians playing instruments like the saxophone, flute, piano, and guitar (Seibel 1962). Chord execution involves the simultaneous depression of several keys to produce one (e.g., on a saxophone and flute) or a combination of tones (piano, guitar). The presently used chord task involved participants simultaneously depressing one, two or three keys of a computer keyboard in response to one, two, or three concurrent key-specific stimuli on the computer display while the fingers were already resting on or slightly above these keys. Below, four mechanisms are discussed that seem involved in executing chords. These mechanisms may be simultaneously involved but their contribution may also differ across skill levels. They involve two different ways of representing chords, (a) at the motor level by way of the required hand postures and (b) at the central-cognitive level as spatial representations. In addition, chord execution may be influenced by (c) the occurrence of biomechanical and/or neural interactions between, especially, neighboring fingers, and in the case of bimanual chords also by (d) the coordination of both hands.

### Posture learning

There is already quite some evidence that movement control involves the use of prestored body postures. This has been demonstrated in a series of behavioral studies (Rosenbaum et al. 1995; Rosenbaum et al. 2001; also see Smyth and Pendleton 1989). That hand postures underlie chording was suggested also by the faster depression of two keys by the fingers of one hand as opposed to two hands (Thon and Bonneviale 1995). Following four experiments with various 2- and 3-element chords practiced for 16 to 40 trials each, Hazeltine et al. (2007) argued that practiced chords are response-specific and do not benefit novel chords. Their data further suggested that after limited practice chord skill does not rely on the learning of stimulus patterns, spatial configurations, or explicit knowledge.

These behavioral indications for motor control on the basis of body postures are consistent with neurophysiological findings. Electrical micro-stimulation applied to motor cortical areas of rhesus macaques and humans for the time such movements typically last yielded movements like bringing the hand to the mouth, reaching to grasp, and various defensive actions (Gentner and Classen 2006; Graziano et al. 2002; Overduin et al. 2014; Overduin et al. 2012). These cortical representations appeared stable in individuals (Ejaz et al. 2015) but highly variable across individuals (Schieber 2002; Schieber and Hibbard 1993), probably because they are shaped by everyday usage (Ejaz et al. 2015). The development of such complex, integrated movement patterns has been attributed to the development of horizontal connections within primary motor cortex (M1) that allow coordinating activity among cortical assemblies that control the individual effectors (Battaglia-Mayer and Caminiti 2019). Novel actions would be executed by involving a broader range of brain areas than just primary motor cortex (Diedrichsen et al. 2013; Ehrsson et al. 2002).

Electrical stimulation of the cortical hand areas has been found to produce “complex postures of the wrist and fingers that resemble[d] typical actions during the manipulation of objects” (p123, Graziano 2016). However, individual fingers are poorly represented in M1 in that their neural representations are highly overlapping (Indovina and Sanes 2001; Wiestler et al. 2011) and M1 neurons are not tuned to control individual finger movements (Acharya et al. 2008). This is in line with the current notion that the skilled control of the human hand with its 34 muscles and its 27 degrees of freedom (Agur and Dalley 2009) is controlled by way of innate or learned hand posture representations. So, even the simple act of pressing a single key with one finger might involve activation of one of a large range of innate or learned hand postures available in primary motor cortex. With movement sequences control is assumed to be initially based on central-symbolic representations and is then gradually taken over by motor representations (Verwey et al. 2015). In a similar vein, one might wonder whether executing chords is also initially controlled using a cognitive—most likely spatial—representation which is then gradually overruled by applying learned hand posture representations.

### Spatial representations

Research has shown that spatial coding is pervasive throughout the brain and supports many cognitive processes (e.g., Groen et al. 2022), including the control of motor sequences (Verwey et al. 2015). Indeed, indications have been reported for close interactions between frontal and parietal areas during movement (Graziano 2016). In the case of chord tasks spatial coding may be used to represent locations of the keys of a chord. A classic study in this respect was reported by Seibel (1963). Across a period of several months, 3 participants practiced for 75 trials each of the 1023 chords that are possible with 10 fingers. The finding that even after this amount of practice RT increased with the number of fingers in a chord seems inconsistent with the use of hand postures and suggests that chording involves a central-symbolic, possibly spatial, representation that is serially read to determine each of the required fingers. This serially readout is consistent with the finding that in the case of 2-finger chords carried out with all finger pairs of the two hands, in a 2-choice RT task the rightmost and in a simple-RT task the leftmost finger was fastest (MacKenzie 1985).

Notions about hand posture learning led to the idea that entering text with QWERTY keyboards might be improved by simultaneously pressing keys on a so-called chord keyboard (Gopher et al. 1985; Gopher and Raij 1988). Cognitive processing was argued to be less demanding than with traditional typing when a chord keyboard is used on which each finger always presses the same key and the letter is determined by the combination of keys pressed. While these chord typewriters have never become a serious challenge to QWERTY keyboards, this research did show high execution and learning rates (Gopher and Raij 1988). Support for the development of spatial, hand-independent representations with extended practice was that typing performance was better when the two key panels had the same spatial lay out for each hand (e.g., when in one plane the leftmost two keys of each panel produced the same letter) than when the two panels had a mirror layout (e.g., the index and middle fingers of each hand produced the same letter) (Gopher et al. 1985). Chording on the basis of spatial representations is in line with involvement in the control of hand postures of the inferior parts of the parietal cortex (Buxbaum 2001; Osiurak and Badets 2017) as this area is responsible for spatial learning.

Further support for the importance of spatial chord coding is that in choice RT tasks S-R compatibility effects depended on the relationship between stimulus and response locations irrespective of the effectors used (Wallace 1971). Additional support for the use of spatial response coding comes from research with choice RT tasks in which one of four keys was pressed by one of four fingers of the two hands and the effect was assessed of precuing two responses (Miller 1982). Responses appeared fastest with left–right cues (locations 12 and 34 when counting from left to right), intermediate with inner-outer cues (14 and 23), and slowest with alternate cues (13 and 24) (Reeve and Proctor 1990). Importantly, this advantage of the left–right cues over the inner-outer and alternate cues disappeared with four fingers of a single hand (Adam et al. 2003; Proctor and Reeve 1986). These findings are consistent with spatial response coding gradually being accompanied by motor coding (Proctor and Reeve 1988). The Grouping Model (Adam et al. 2005) accounts for such precuing effects by the notion that visual displays consisting of multiple elements are organized into visuospatial subgroups and these induce a similar grouping in the response set while motoric factors provide constraints. Together, these results suggest that chords are initially coded in terms of key locations while with practice this is accompanied by a direct relationship between a stimulus pattern and a hand posture representation.

### Interactions between individual fingers

The phenomenon that movements of body parts induce concurrent movement of other body parts has been called enslaving (Zatsiorsky et al. 1998). In the case of chording, some chords may be harder to produce than others because of biomechanical and/or neural interactions between the neighboring fingers of one hand (Hirano et al. 2018; Lawrence and Hopkins 1976; Zatsiorsky et al. 2000). Those fingers may be enslaved biomechanically by the anatomical connection of tendons across fingers, and neurally by the synchronized activity of motor units in different compartments of the multi-tendoned flexors and extensor of the digits (Leijnse et al. 1993) and by the overlapping representations of individual fingers in the motor cortex (Furuya et al. 2014). These more or less structural interactions occur especially between the middle and ring fingers and also the pinky, while the thumb and index finger can move virtually independently (Van Den Noort et al. 2016; Zatsiorsky et al. 2000).

Research showed that beginning pianists gradually learn to overcome interactions between the fingers (Furuya et al. 2014), and that after lifelong practice professional pianists achieve almost full independence of all fingers (Furuya et al. 2011). Before this independence is achieved, people may learn to adjust to the anatomical constraints (Leijnse et al. 1993) using hand postures with minimal finger position differences (Van Den Noort et al. 2016). Such learning may well involve cortically mediated inhibition of enslaved finger movements (Aoyama et al. 2019).

### Bimanual chords

It is not clear whether chords that involve fingers of both hands are controlled by a single representation or by two hand-specific representations. Development of a single chord representation that encompasses postures of both hands might be beneficial because that reduces the need for cognitively loading coordination of both hands (Heuer 1995; Spijkers et al. 2000; Thon and Bonneviale 1995).

Development of bimanual chord representations was suggested after just 16 practice trials per chord (Hazeltine et al. 2007). However, the finding that bimanual chords were executed slower than unimanual chords suggests another interpretation. Instead of executing a single bimanual chord representation, participants may have selected two unimanual postures in succession after which a bimanual coordination process allowed simultaneous depression of all keys. This interpretation is consistent with indications that the faster hand awaited the slower hand (MacKenzie 1985), and the conclusion that preparing 2 fingers in unimanual chords occurred simultaneously while preparing two (non-homologues) fingers of two hands occurred in rapid succession (Thon and Bonneviale 1995).

Neurophysiological findings seem consistent with bimanual chord representations. While traditionally each hand was believed to be controlled solely by the contralateral primary motor cortex (see, e.g., Whitehead and Banihani 2014), research on motor sequences and chords demonstrated that the ipsilateral Ml is involved in finger movements too (Chen et al. 1997; Grafton et al. 2002; Verstynen et al. 2005). This was most obvious in right-handers but occurred in most left-handers too (Verstynen et al. 2005). These findings indicate that both hemispheres are involved in controlling each hand which probably allows a single chord representation that controls postures of both hands.

### The present study

To understand how individual fingers are controlled when, for example, playing musical instruments and grasping peculiarly shaped objects, the present study investigated the processes responsible for the skill to produce chords with four fingers of either one or two hands. The above literature review showed that several mechanisms may underlie the representation and the execution of chording movements. The spatial chord hypothesis assumes that chord skill is based on a representation of the key locations (Gopher et al. 1985) and predicts that chord skill can transfer to other fingers. The general posture hypothesis posits that a single posture representation encompasses unimanual as well as bimanual chords and that using this representation is equally fast with one and with two hands because both hands are prepared simultaneously. In contrast, the hand-specific posture hypothesis posits that chord learning involves separate posture representations for individual hands that are prepared one after the other. Both posture hypotheses imply that chord learning is effector-specific and does not transfer to other fingers, but they differ as to whether in bimanual chording the two hand postures are prepared concurrently or successively, which would show when comparing RTs of unimanual and bimanual chords.

The spatial chord hypothesis further predicts that RT increases with the number of keys in a chord, irrespective of whether one or two hands are used, because the individual key locations are successively read from a spatial representation in memory (Sternberg 1966). The two posture hypotheses basically suggest that the hand posture is selected as whole, irrespective of the number of fingers involved. However, all three hypotheses take into account that the implementation of a selected chord representation is slower as more fingers are involved because of the higher (biomechanical and neural) interference between adjacent fingers. This enslavement of individual fingers implies that the expected RT increase for chords with more keys is more limited when the same chords are being executed with two than with one hand. Nonetheless, bimanual chord execution may also be slower because the hands need to be coordinated (Heuer et al. 1998; MacKenzie 1985). Such coordination could involve the successive preparation of the two hand postures while the hand prepared first awaits preparation of the other hand before both hands simultaneously press their keys.

Transfer of chord skill to other fingers and the effect of number of keys in a chord, were tested by having participants practice two chords consisting of 3 simultaneous key presses, two chords of 2 key presses, and two single key presses in response to the simultaneous filling of 3, 2, or 1 of the four horizontally displayed placeholders. Each of these 6 responses was practiced for 240 trials. The Right-hand group carried them out with the four right-hand fingers (no thumb). The 2-Hand group practiced the responses using the index and middle fingers of both hands. The keys used in each chord were counterbalanced across participants to make sure that the expected number-of-fingers effect was independent of the specific fingers used and their interactions. Single-key responses were included to see whether the number-of-fingers effect occurred in 2-key chords too.

The effect of inter-finger interference (i.e., enslavement) was explored with the newly developed Chord Complexity Index (CCI). This CCI indicates for each chord the total number of adjacent fingers that move differently and this is assumed to approximate total enslavement in a chord. The test phase involved practiced and novel chords executed with either the practiced hand configuration or the hand configuration of the other practice group. As previous work showed that participants had little awareness of the chords after 16–40 practice trials (Hazeltine et al. 2007), the present study examined whether perhaps after 240 practice trials per chord the participants would be more aware of the keys they had been depressing during practice and whether they would actually use that knowledge for chord execution.

## Methods

### Participants

Thirty-two students participated in exchange for course credits (mean age 21.5 years old, s.d. 2.6 years). This number was determined by counterbalancing chords which yielded 16 different chord combinations for each participant group. Participants were allocated to one of the two participant groups on the basis of their participation order (see Appendix A). One participant was replaced because of the excessive number of errors. All participants indicated to be right-handers, non-smokers (to prevent distractive withdrawal symptoms) and not to have used alcohol in the preceding 24 h. The experiment was approved by the faculty of Behavioural, Management and Social Sciences (BMS) ethics committee. The participants signed an informed consent before the experiment.

### Apparatus

The experiment was programmed and conducted in E-Prime 2.0 running under Windows 10. Instructions and stimuli were presented on a 24 inch AOC Freesync monitor with a 144 Hz refresh rate. Participants used the C, V, B and N keys of a standard PS/2 Logitech Deluxe 250 keyboard (Logitech, Newark, CA, USA). A PS/2 keyboard was used because it is better able to detect simultaneous key press than the nowadays more common USB keyboards. Low level E-Prime script, in combination with the 144 Hz monitor and the PS/2 keyboard, allowed millisecond (ms) accuracy of simultaneously pressed keys. During the experiment, unnecessary programs and Windows services were turned off to improve RT measurement. Participants were given the opportunity to use keyboard wrist resting pads for one or both hands, and most participants actually used them. The room (2.25 × 2.25 × 3.50 m) in which the participant performed the experiment was dimly lit and was equipped with a video camera for participant monitoring.

### Task

**Fig. 1:**
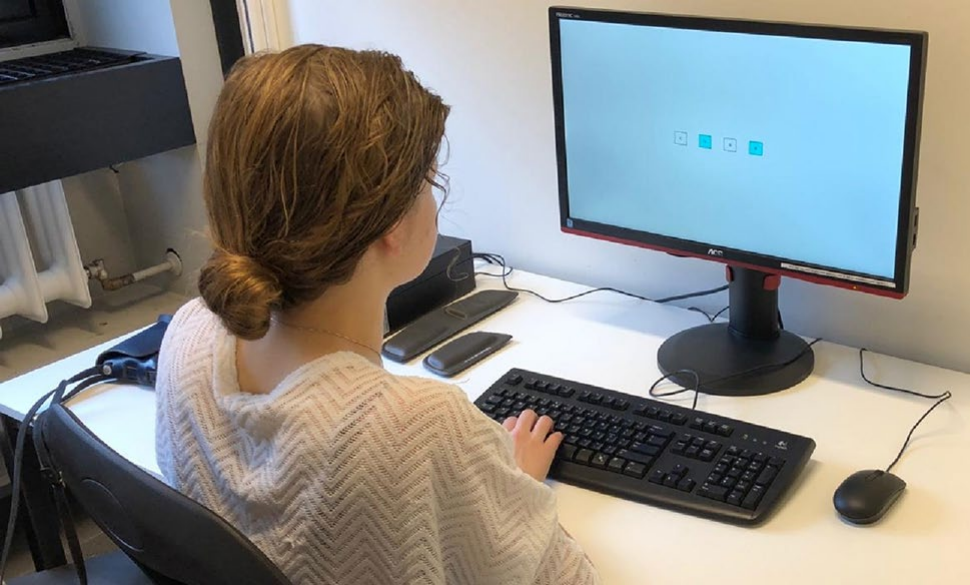
A participant of the Right-hand group executing a 2-key chord

Odd numbered participants made up the Right-hand group and used their right index, middle, ring and little fingers to press the C, V, B and N keys on the QWERTY keyboard. Even numbered participants formed the 2-Hand group and used the index and middle fingers of both hands to press the same keys (see the overview in Appendix A). At the start of each trial block instructions were displayed which fingers and hands to use. During each block, the display permanently showed a row of four black 12 × 15 mm placeholders at mutual distances of 37 mm in the center of a white screen. At the center of each placeholder, the letters “C”, “V”, “B” and “N” were displayed from left to right, showing the associated key (Fig. 1). Stimuli consisted of simultaneously filling 1, 2 or 3 placeholders with cyan, indicating the key(s) to press. Stimuli were displayed for 2000 ms during which the participants responded. Next, feedback was displayed for 1000 ms. This involved the cyan fillings being replaced with silver fillings. In the case of an error this was accompanied by displaying an error message: if the time between the first and last key press of a multi-element chord exceeded 50 ms then ‘timing error’ was displayed in red over the four placeholders. If a key had inadvertently not been pressed ‘miss’ appeared in red below that placeholder. And if an incorrect key had been pressed ‘false’ appeared in red below the relevant placeholder(s). After this 1000 ms feedback period, error messages were erased and the placeholders were again filled with the white background color. Then the nonaging 500 to 2000 ms interstimulus interval started that was followed by display of the next stimulus.^1^

Practice included 8 practice blocks, each including 180 trials. Participants practiced a choice task with 6 different responses consisting of two three-key chords (2-Hand group: one with 1 left hand finger and 2 right-hand fingers, the other with 2 left hand fingers and 1 right-hand finger), two two-key chords (2-Hand group: one involving 2 fingers of 1 hand, the other one finger of either hand), and two one-key responses (2-Hand group: one with a finger from each hand). As shown in Appendix A, the available chords were counterbalanced across the practice and test phases across participants. By the end of practice, each of the 6 chords had been practiced for 240 trials. During each practice block, a chord was drawn randomly without replacement from the set of 6 alternative chords.

The test phase consisted of four separate 42-trial subblocks that together involved a 2 (chord familiarity) by 2 (hand configuration) design. Their order was counterbalanced across the participants. So, the practiced and novel chords were carried out in separate blocks, either with the same hand configuration as during practice (4 right-hand fingers or two fingers of each hand), or with the hand configuration of the other practice group. The practiced chords included 6 different chords. The novel chords consisted of 6 comparable chords that were new to the participant but practiced by other participants.

### Procedure

The experimenter welcomed each participant, checked whether they met the participation criteria and introduced the experimental task. Then the participant was taken to the cubicle and instructed about the task.

The first 8 blocks of trials constituted the practice phase. Each block included two 90-trial subblocks with the 6 different chords. The first of these blocks started with written instructions on the screen including the instruction what fingers to use. The first subblock was followed by a 20 s break after which the next subblock started. The second subblock was followed by a 5 min break. Both breaks involved a counter counting down the seconds to zero. At the end of the long break, the experimenter entered the cubicle, started the next block, and left the room.

After practice the experimenter introduced the questionnaire and remained in the cubicle while it was filled out. After filling out questions about demographics, age, smoking and alcohol consumption participants were asked to write down the chords they had been practicing in terms of the keys pressed. This part of the questionnaire constituted the awareness test. Participants had not been told that awareness would later be tested. The awareness test involved a drawing with the positions of the four keys to allow them to know which finger was associated with what key. Finally, they responded to a multiple-choice question how they had recalled the chords in which they were allowed to check more than one option. These options were (a) I remembered the order of the letters. (b) I remembered the positions of the keys. (c) I remembered the positions of the squares on the screen. (d) I pressed the keys in my mind. (e) I pressed the keys on the tabletop. Lastly, the participant could write down remarks on the form. Then the test phase in Block 9 followed which consisted of the 4 subblocks.

### Data analyses and design

The main dependent variable was chord reaction time (RT) of accurate chords. It consisted of the mean of the times taken to depress each of the keys relative to the onset of the key-specific stimuli on the display. Chord timing was accurate only when the fastest and slowest key presses did not differ more than 50 ms. The Chord Complexity Index (CCI) was developed to quantify interference between adjacent fingers. It involves counting for each finger whether it behaves differently from 0, 1 or 2 of its neighboring fingers of the same hand. These numbers were then summed across all fingers for each chord (Appendix B). For example, a unimanual chord involving a key press by just the ring finger yielded a CCI of 4 because the ring finger has 2 differently moving neighbors (the middle finger and the pinky) and the middle finger and the pinky each have 1 differently moving neighbor (namely the ring finger). A unimanual chord involving the index, ring and little finger yielded a CCI of 1 + 2 + 1 + 0 = 4. And a bimanual chord depressing the two left and not the two right keys yielded a CCI of 0. CCI was higher for the Right-hand group than for the 2-Hand group, 3.2 vs. 2.0.

Statistical significance was assumed with p-values smaller than *α* = 0.05. The *p* values of the *F* tests were Greenhouse–Geisser corrected when Mauchley's test of sphericity was significant. Error proportions were subjected to an arcsine transformation and then analyzed with the same ANOVA designs as RTs (Winer et al. 1991). Most ANOVAs and contrast analyses were carried out with Statistica but the more advanced CCI analyses were carried out with the Afex package in R.

## Results

### Practice phase

#### Reaction times

A 2 (Practice Group: Right-hand vs. 2-Hand) × 3 (Number-of-Fingers: 1, 2, or 3 keys) × 2 (Repetition) × 8 (Block) ANOVA was used to analyze the RTs with Practice Group as between-subject variable. Repetition covered effects caused by differences between the chord pairs with the same number of keys and these chords were analyzed in more detail in a separate CCI analysis that is reported at the end of this section.

**Fig. 2:**
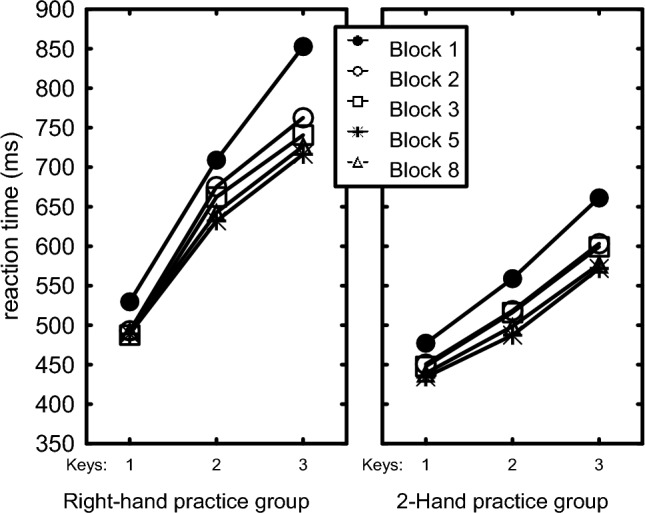
RT as a function of number of keys in the chord and practice block. Blocks 4, 6 and 7 and standard errors of the means are omitted for clarity

The ANOVA showed a main effect of Block, *F*(7,210) = 23.90, *p* < 0.001, *η*_p_^2^ = 44, and of Practice Group, *F*(1,30) = 20.17, *p* < 0.001, *η*_p_^2^ = 0.40, indicating that RTs reduced across successive blocks, and that chords were faster with two hands than with the right hand (515 ms vs. 631 ms). The Chord main effect showed that RT increased as a chord included more keys, *F*(2,60) = 125.86, *p* < 0.001, *η*_p_^2^ = 0.81. According to the Chord x Practice Group interaction, *F*(2,60) = 10.62, *p* = 0.003, *η*_p_^2^ = 0.26, this RT increase with more keys was more pronounced for the Right-hand than for the 2-Hand group (Fig. 2). A Chord x Block interaction, *F*(14,420) = 10.02, *p* = 0.004, *η*_p_^2^ = 0.25, showed that the RT increase with number of fingers reduced with practice. Planned comparisons showed that across both practice groups the RT reduction from Block 1 to Block 2 was larger for the 2- and 3-key chords than for the 1-key response, *F*(1,30) = 9.2, *p* = 0.005, *η*_p_^2^ = 0.23, while it was not different for the 1-key response and just the 2-key chords, *F*(1,30) = 0.65, *p* = 0.43. The indication in Fig. 2 that improvement of 3-key chords was larger between Blocks 1 and 2 in the Right-hand than the 2-Hand group was marginally significant, *F*(1,30) = 3.54, *p* = 0.07, *η*_p_^2^ = 0.11.

A similar ANOVA on the last practice block, Block 8, confirmed by way of a Practice Group main effect that in Block 8 the 2-Hand group was still faster than the Right-hand group (505 vs. 621 ms), *F*(1,30) = 26.34, *p* < 0.001, *η*_p_^2^ = 0.47, and that chords with more fingers were still executed more slowly (466 ms, 571 ms, and 652 ms, resp.), *F*(2,60) = 132.94, *p* < 0.001, *η*_p_^2^ = 0.81. The Practice Group x Chord interaction, *F*(2,60) = 10.95, *p* < 0.001, *η*_p_^2^ = 0.27, confirmed that the benefit of 2-Hand over Right-hand participants was larger for the 2-and 3-key chords (benefits: 144 and 149 ms, resp.) than for the 1-key response (54 ms). The 2-Hand benefit caused by pressing individual keys with fingers of different hands was significant for the single key presses too, *F*(1,30) = 11.61, *p* = 0.002, *η*_p_^2^ = 28.

In summary, RTs were longer for the Right-hand than for the 2-Hand group, RTs increased with the number of keys in a chord and more so for the Right-hand than the 2-Hand group. These effects reduced with practice but were still significant in the last practice block.

#### Errors

Arcsine transformed proportions of the sums of timing and accuracy errors in the practice blocks were analyzed with the same 2 × 3 × 2 × 8 ANOVA as the RTs. A Number-of-Fingers x Practice group x Block interaction, *F*(14,420) = 4.76, *p* < 0.001, *η*_p_^2^ = 0.14, showed more errors in the Right-hand than in the 2-Hand group for the 3-key chords and that this difference reduced with practice. In Block 1, error rate for the 1-, 2- and 3-key chords amounted to 0.5%, 10.1% and 34.4%, respectively, for the Right-hand group, and 0.6%, 3.5%, and 11.7% for the 2-Hand group. In Block 8, these percentages had reduced to 0.7%, 4.0%, and 8.5% for the Right-hand group and 0.6%, 1.3% an 5.0% for the 2-Hand group. This effect contributed to a general error reduction of practice (from 10.1% in Block 1 to between 3.9% and 3.3% across Blocks 4 through 8), *F*(7,210) = 30.88, *p* < 0.001, *η*_p_^2^ = 0.18, and to Right-hand practice generally yielding more errors than 2-Hand practice (6.7 vs. 2.8%), *F*(1,30) = 10.83, *p* = 0.002, *η*_p_^2^ = 0.27. The Practice Group x Block interaction, *F*(7,210) = 6.67, *p* < 0.001, *η*_p_^2^ = 0.18, showed that error rate reduced especially for the Right-hand group, from 15.0% in Block 1 to 4.4% in Block 8 and less so for the 2-Hand group for which it reduced from 5.3% in Block 1 to 2.2% in Block 8. Planned comparisons showed that the 2-Hand group had less errors than the Right-hand group in Block 8 too, *F*(1,30) = 5.57, *p* = 0.02 *η*_p_^2^ = 0.16.

The Number-of-Fingers main effect showed that error rate increased with the number of keys in a chord (0.6%, 3.0%, and 10.7% resp.), *F*(2,60) = 54.40, *p* < 0.001, *η*_p_^2^ = 0.64, which effect reduced with practice as shown by a Block x Number-of-Fingers, *F*(14,420) = 18.66. *p* < 0.001, *η*_p_^2^ = 0.38. Error rate for the 1-, 2-, and 3-key chords went from 0.6%, and 6.8%, to 23.0%, respectively, in Block 1 to 0.7%, 2.6% and 6.7% in Block 8, respectively. The Number-of-Fingers effect was still significant across both practice groups in Block 8, *F*(2,60) = 20.07, *p* < 0.001, *η*_p_^2^ = 0.40. The higher error rate for chords with more fingers also reduced with practice as shown by a Number-of-Fingers x Practice group interaction, *F*(2,60) = 7.56, *p* = 0.007, *η*_p_^2^ = 0.20. Error rate with 1-key chords was the same for the Right-hand and 2-Hand practice groups (both 0.6%), was larger for the Right-hand than the 2-Hand group with 2-key chords (4.2 vs. 1.8%, respectively) and 2-Hand advantage increased even more with 3-key chords (15.3 vs. 6.1%, respectively).

In short, the proportion of errors in accuracy and timing was higher for the Right-hand than for the 2-Hand practice groups, for chords with more keys, and it was especially high for the Right-hand group producing 3-key chords. The higher error rates in the Right-hand group and in chords with more fingers reduced with practice but were still statistically significant in Block 8.

#### CCI analyses

**Fig. 3:**
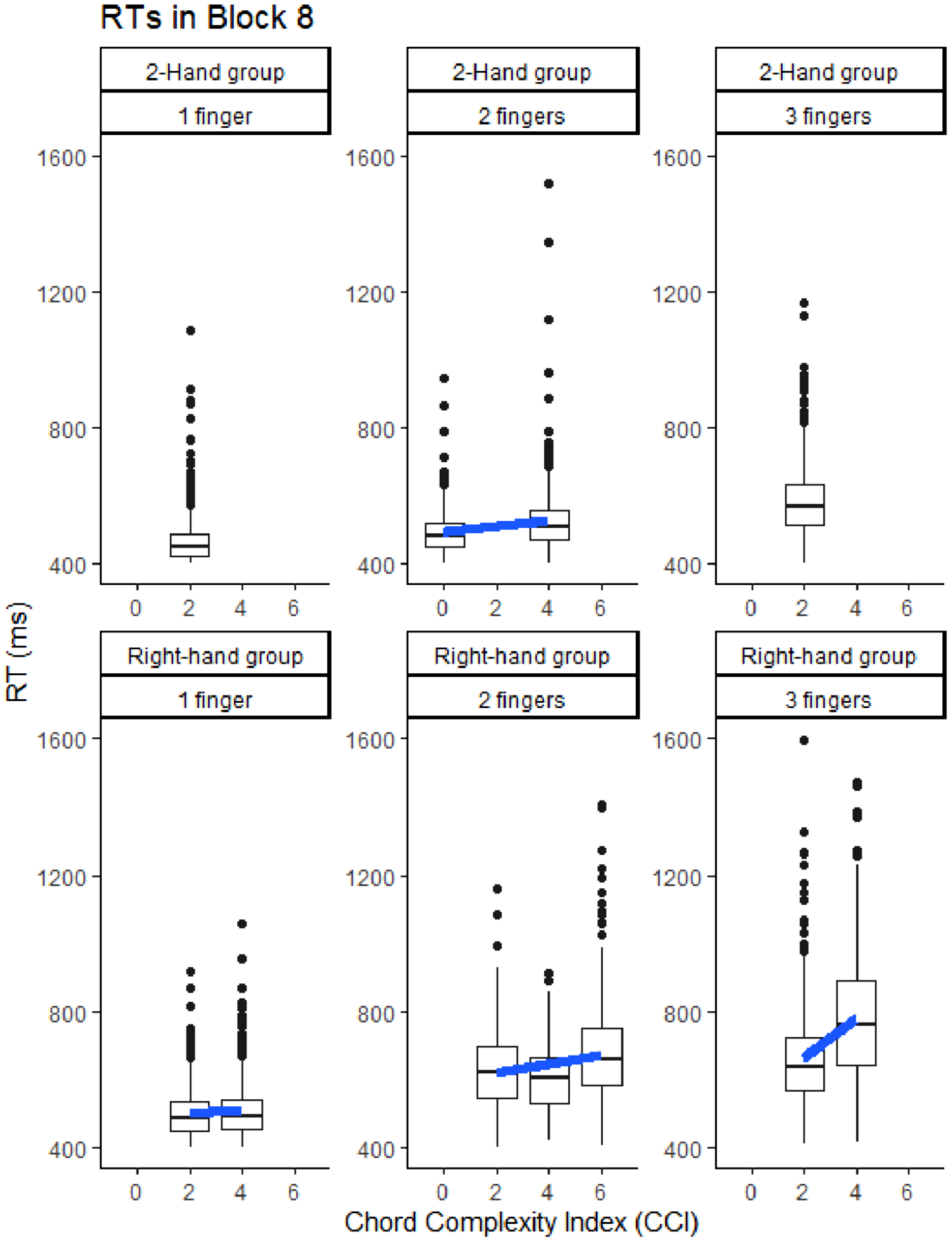
RTs to chords involving 1, 2 and 3 fingers in Block 8 across all participants of the two practice groups along with the regression lines in chords with different CCIs

Counterbalancing chords implied that for subsets of participants their two practiced chords with a particular number of fingers had different CCIs. This held for all 2-Hand participants in the case of 2-finger chords, and for some Right-hand participants in the case of 1-, 2- and 3-finger chords. This allowed preliminary tests within participants of the hypothesis that higher levels of interference between adjacent fingers—indicated by greater CCIs—slows chords independent from the number of fingers in these chords. Figure 3 shows the RTs per chord as a function of CCI for various chords at the end of practice in Block 8. Table 1 presents planned comparisons of the effects of CCI on RTs for the various chords carried out by both practice groups in Blocks 1 and 8.

**Table 1:** RTs and significance levels of planned comparisons for chords in the two practice groups with different CCIs for chords with a fixed number of fingers in Blocks 1 and Block 8 (cf. Figure 3)

Group	Number of fingers	CCI-Chord	RTs in Block 1 with the CCIs indicated	RTs in Block 8 with the CCIs indicated	CCI x Block (1 vs. 8)
2-Hand	2 (*n* = 16)	CCI-0 (CVxx xxBN) CCI-4 (CxBx CxxN xxBN)	520 vs. 584 ms***	474 vs. 522 ms*	^*ns*^
Right-Hand	1 *(n* = 8)	CCI-2 (Cxxx or xxxN) CCI-4 (xVxx or xxBx)	509 vs. 553 ms*	501 vs. 485 ms ^ns^	*
	2 (*n* = 5)	CCI-2 (CVxx xxBN) CCI-4 (CxxN)	622 vs. 633 ms^ns^	599 vs. 606 ms ^ns^	^*ns*^
	2 (*n* = 11)	CCI-2 (CVxx xxBN) CCI-6 (CxBx xVxN)	673 vs. 785 ms**	631 vs. 682 ms ^ns^	**
	3 (*n* = 8)	CCI-2 ( CVBx xVBN) CCI-4 (CVxN CxBN)	728 vs. 903 ms**	671 vs. 782 ms**	^*ns*^

Also indicated are the numbers of participants involved in each comparison

***p < .001; **p < .01; *p < .05; ^ns^*p* > .05

Table 1 shows that RTs in Block 1 were slower as CCI was larger in chords with the same number of fingers, except for the 2-finger chords of Right-hand participants with CCIs 2 and 4. For two chords, the effect of CCI on RTs did not change with practice while in the three others the CCI effect had disappeared in Block 8. In the 2-Hand participants executing 2-finger chords with 1 hand (CCI-0) was faster in Blocks 1 and 8 than chords involving a finger of both hands (CCI-4). Similarly, Right-hand participants executing chords in Blocks 1 and 8 with 3 adjacent fingers (CCI-2) was faster than 3-finger chords with a deviating finger in between the other fingers (CCI-4). Two still different chords showed that the CCI effect vanished with practice. This happened when Right-hand participants pressing in Block 1 a key at the far left and right of the four fingers were faster than when they pressed one of the middle two keys. This RT difference had disappeared in Block 8. In Block 1, Right-hand participants pressing the left two and the right two keys (CCI-2) were faster than a 2-key chord with alternating keys (CCI-6) but not faster than pressing the outer two keys (CCI-4). This disadvantage of the alternating keys had disappeared in Block 8.

Further planned comparisons showed that the larger RTs as there were more fingers in a chord was significant also in chords with the same CCI. This number-of-fingers effect reduced significantly with practice but was still significant in Block 8. This was demonstrated by three planned comparisons. (a) The first one involved the 2-Hand group executing 1- and 3-finger chords with a CCI of 2 in Block 8. It showed that 1-finger key presses were executed faster than 3-finger chords (439 vs. 575 ms), *F*(1,15) = 96.41, *p* < 0.001, *η*_p_^2^ = 0.87. This 136 ms benefit of 1- over 3-finger chords in Block 8 was smaller than the 179 ms benefit in Block 1, *F*(1,15) = 5.50, *p* = 0.03, *η*_p_^2^ = 0.27. (b) In the Right-hand group, RTs obtained in Block 8 showed that across the 9 participants with CCI-2 in 1-, 2- and 3-key chords, RT increased with number of fingers (1-finger: 498 ms, 2-fingers: 633 ms, 3-fingers: 662 ms), *F*(2,16) = 25.85, *p* < 0.001, *η*_p_^2^ = 0.76. This effect was significant in Block 1 as well (484 ms, 664 ms, 754 ms, respectively), *F*(2,16) = 29.33, *p* < 0.001, *η*_p_^2^ = 0.79, and this effect of number of fingers reduced more with practice as the chords involved more fingers (by -14 ms, 31 ms, 91 ms, respectively), *F*(2,16) = 6.12, *p* = 0.01, *η*_p_^2^ = 0.43. Finally, (c) across the 3 Right-hand participants executing 1-, 2- and 3-key chords with CCI-4, RT also increased with fingers in the chord (507 ms, 648 ms, 854 ms, respectively), *F*(2,4) = 40.15, *p* = 0.002, *η*_p_^2^ = 0.95. Here, too, the effect was observed also in Block 1 (594 ms, 680 ms, 1042 ms, respectively), *F*(2,4) = 84.29, *p* < 0.001, *η*_p_^2^ = 0.98, and tended to reduce more with practice as the chord included more fingers (by 87 ms, 32 ms, 188 ms, respectively), *F*(2,4) = 6.04, *p* = 0.06, *η*_p_^2^ = 0.75.

An indication that the benefit of 2-Hand over Right-hand participants is not only caused by inter-finger interference is that 2-Hand participants executed CCI-2 chords faster than Right-Hand participants in Block 1 (561 vs. 621 ms), *F*(1,30) = 4.97, *p* = 0.03, *η*_p_^2^ = 0.14, and in Block 8 (505 vs. 590 ms), *F*(1,30) = 14.77, *p* < 0.001, *η*_p_^2^ = 0.33. This 2-Hand group benefit was similar in Blocks 1 and 8, *p* = 0.53.

In short, the results confirmed that RTs increased in chords with higher CCIs while the number of keys remained the same, and showed that this CCI effect had vanished in Block 8 in some but not in other chords. Chords were also executed slower as the number of fingers in a chord increased even when CCI was fixed. This number-of-fingers effect reduced with practice, especially for 3-key chords, but it had not disappeared in Block 8. Finally, the 2-Hand over Right-hand benefit was observed even in chords with the same CCI of 2.

### Test phase

#### Reaction times

RTs in the test block were analyzed with a 2 (Practice Group) × 3 (Number-of-Fingers) × 2 (Hand Configuration: familiar vs. unfamiliar) × 2 (Chord Familiarity: practiced vs. novel) × 2 (Repetition) mixed ANOVA with Practice Group as between-subject variable. Below, indications in the RT analyses for chord learning and coordination learning in bimanual chording are separately reported. This is followed by the error analysis.

#### Chord-specific learning

**Fig. 4:**
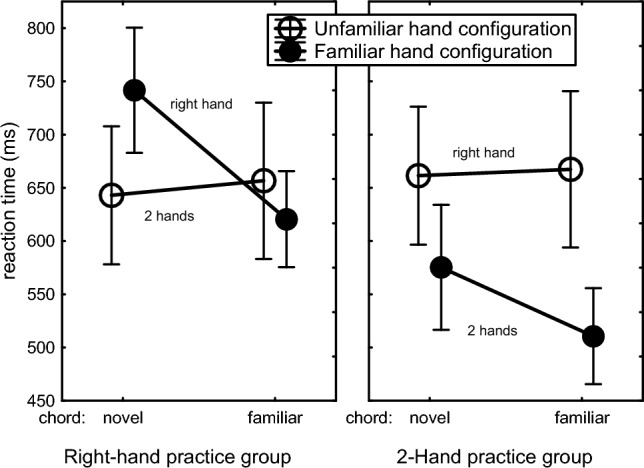
Reaction times in the test phase across 1-, 2-, and 3-finger chords in the two practice groups as a function of used hand configuration and chord novelty. Here and in the figures below, the bars indicate the standard error of the means

The Hand Configuration x Chord Familiarity interaction, *F*(1,30) = 21.22, *p* < 0.001, *η*_p_^2^ = 0.41, showed chord-specific learning in that, across both practice groups, the practiced chords were executed faster by the familiar than by the unfamiliar hand configuration (566 vs. 658 ms, respectively), *F*(1,30) = 36.91, *p* < 0.001, *η*_p_^2^ = 0.55, whereas there was no benefit of the familiar hand configuration when executing novel chords (652 vs. 662 ms), *F*(1,30) = 0.16, *p* = 0.69. This interaction was reflected also in the Chord Familiarity main effect, *F*(1,30) = 14.58, *p* < 0.001, *η*_p_^2^ = 0.33, and the Hand Configuration main effect, *F*(1,30) = 16.88, *p* < 0.001, *η*_p_^2^ = 0.36. Importantly, planned comparisons showed that this Hand Configuration x Chord Familiarity interaction, indicative for chord-specific learning, was significant for each practice group, *F*s(1,30) > 5.01, *p*s < 0.04, *η*_p_^2^ > 0.14 (Fig. 4).

**Fig. 5:**
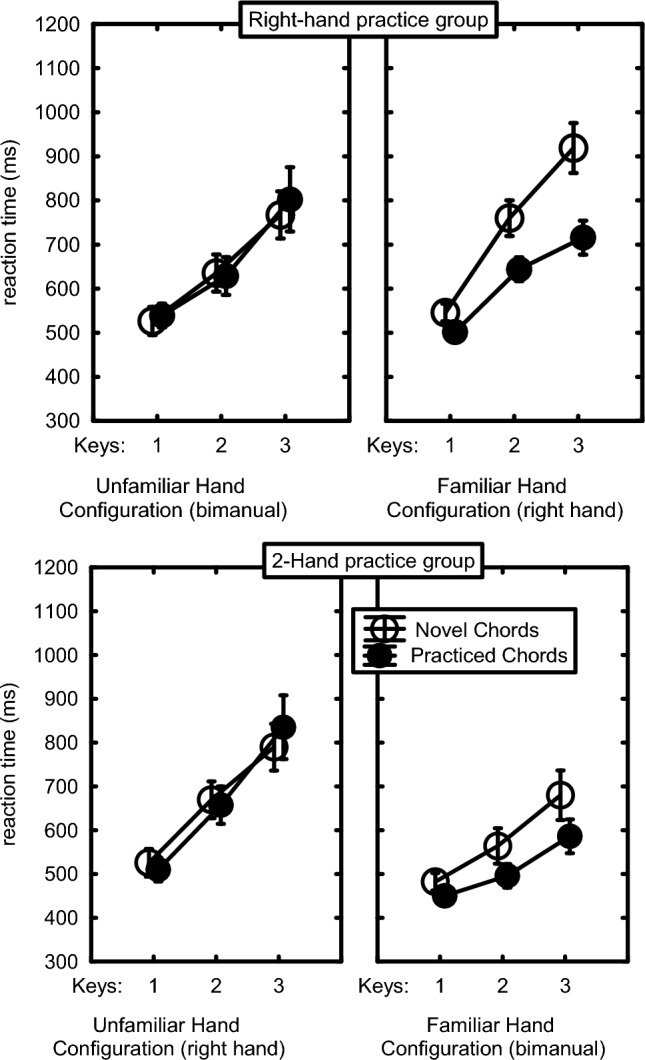
Reaction times in the test phase of practiced and novel chords including 1, 2 and 3 keys as carried out by the Right-hand and the 2-Hand practice groups using the familiar and the unfamiliar hand configuration

The Hand Configuration x Number-of-Fingers interaction showed that the RT increase with more fingers in a chord, as indicated also by the Number-of-Fingers main effect (1 key: 510 ms, 2 keys: 632 ms, 3 keys: 762 ms), *F*(2,60) = 194.61, *p* < 0.001, *η*_p_^2^ = 0.87, was stronger with unfamiliar than with familiar hand configurations, *F*(2,60) = 3.98, *p* = 0.05, *η*_p_^2^ = 0.12. The Chord Familiarity x Hand Configuration x Number-of-Fingers interaction showed that this RT increase with number of fingers in the familiar hand configuration was actually stronger with novel than with practiced chords, *F*(2,60) = 14.43, *p* < 0.001, *η*_p_^2^ = 0.32. As depicted in Fig. 5, this reduced number-of-fingers effect for the familiar hand configuration executing the practiced chords was seen with both practice groups, *F*s(2,60) > 4.64, *p*s < 0.02, *η*_p_^2^ = 0.13, and did not differ for these practice groups, *F*(2,60) = 0.57, *p* = 0.57. In short, in both practice groups the benefit of the familiar hand configuration emerged only with familiar chords. The RT increase with number of fingers was stronger in both practice groups for unfamiliar than familiar hand configurations and for novel than practiced chords.

### Learning between-hand coordination

As indicated by Figs. 4 and 5, the 2-Hand practice group was faster across novel and practiced chords with two hands than with the right hand (543 vs. 664 ms, respectively) whereas the Right-hand practice group was about as fast with two hands as with the right hand (681 vs. 650 ms, respectively). This was endorsed by a Practice Group x Hand Configuration interaction, *F*(1,30) = 48.28, *p* < 0.001, *η*_p_^2^ = 0.62, which was responsible also for the Practice Group main effect (2-Hand group: 604 ms vs. Right-hand group: 665 ms), *F*(1,30) = 4.27, *p* = 0.047, *η*_p_^2^ = 0.12. These findings show that producing chords with two hands benefits more from practice than right-handed chording. Separate tests confirmed that this larger transfer to the unfamiliar hand configuration in the 2-Hand than in the Right-hand practice group was also significant with just familiar and also with just novel chords, *F*s(1,30) > 14.42, *p*s < 0.001, *η*_p_^2^s > 0.32. Hence, in addition to the above indications for hand configuration-specific chord skill, practice also induced chord-unspecific benefits for 2-Hand chording experience. The Practice Group x Hand Configuration x Number-of-Fingers interaction, *F*(2,60) = 15.12, *p* < 0.001, *η*_p_^2^ = 0.34, showed that this effect was stronger with 2- and 3-finger chords than with a single finger.

So, the test block showed that in both practice groups the RT benefit of the familiar hand configuration emerged only with familiar chords and this effect was stronger as more fingers were involved. This indicates that practicing chords improves execution with the same and not with another hand configuration. Furthermore, practicing with two hands yielded an advantage when executing 2-handed chords, even when novel, as compared with participants who had been practicing with their right hand, and this effect was stronger in chords involving more fingers. This indicated that 2-handed practice improved bimanual coordination.

### Errors

**Fig. 6:**
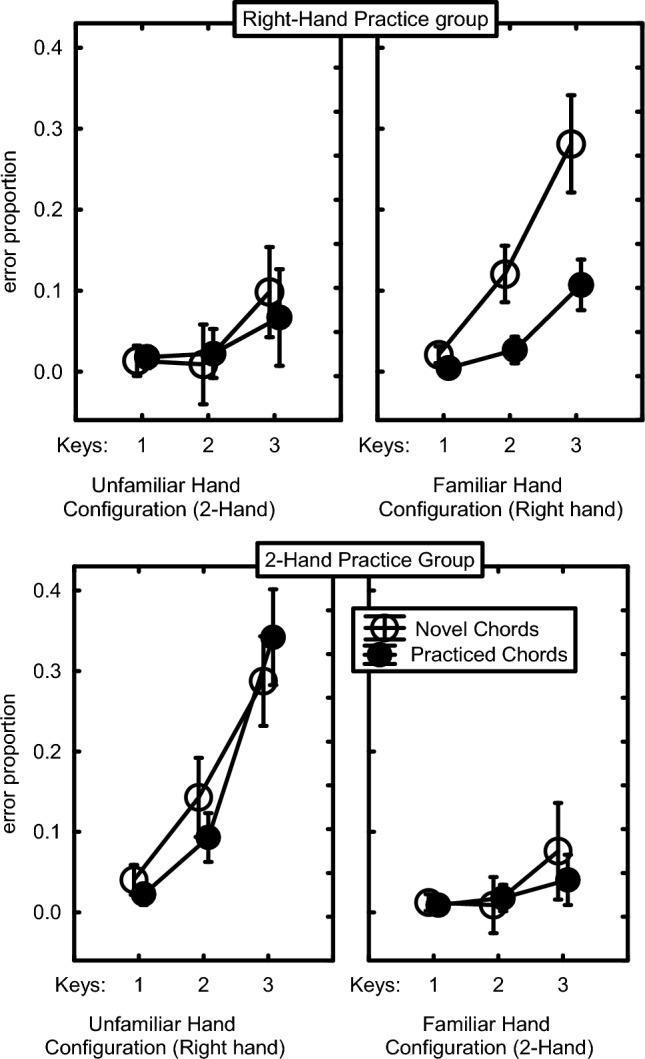
Errors rates in the test phase of practiced and novel chords including 1, 2 and 3 keys as carried out by the Right-hand and the 2-Hand practice groups using familiar and the unfamiliar hand configurations (cf. Fig. 5)

The arcsine transformed proportions per block of chords with errors or poor timing were analyzed with the same 2 × 3 × 2 × 2 × 2 mixed ANOVA as the RTs. The error rates in Fig. 6 show a remarkably similar pattern as RTs in Fig. 5. However, error rate was quite high when the Right-hand group executed novel chords with the right hand and when the 2-Hand group produced practiced and novel 2- and 3-key chords with the right hand. Error rate appeared to increase with the number of keys of the chord (1 key: 1.8%, 2-key: 5.5%, 3-key: 16.2%), *F*(2,60) = 54.49, *p* < 0.001, *η*_p_^2^ = 0.64. Figure 6 shows that in both practice groups the familiar hand configuration produced the practiced chord with error rates similar to those at the end of practice. In contrast, error rate increased in the Right-hand group, especially for the 3-key chords, when the right hand generated novel chords while that group had low error rates when producing the novel and practiced chords with both hands. In a similar vein, the 2-Hand group showed increased error rates when producing practiced and novel (3-key) chords with the right hand while error rate was low with novel and practiced chords produced with both hands. This pattern of results received statistical support from a Practice Group x Hand Configuration x Chord Familiarity interaction, *F*(1,30) = 4.25, *p* = 0.048, *η*_p_^2^ = 0.12, and the superseding Hand Configuration x Chord Familiarity interaction, *F*(1,30) = 5.92, *p* = 0.02, *η*_p_^2^ = 0.16. These interactions confirmed, across all key presses of the chord, that the Right-hand participants had relatively many errors only when novel chords were carried out with the right hand while the 2-Hand participants had more errors when the right hand executed practiced and novel chords. That these effects were moderated by the number of keys per chord is shown by a Practice Group x Hand Configuration x Number-of-Fingers interaction, *F*(2,60) = 29.70, *p* < 0.001, *η*_p_^2^ = 0.50, and the superseding Hand Configuration x Number-of-Fingers interaction, *F*(2,60) = 4.16, *p* = 0.02, *η*_p_^2^ = 0.12. These effects showed that error rate increased with number of keys per chord when the chords were carried out with the right hand in both groups and not when both hands were used. This group difference was not supported by a significant Practice Group x Hand Configuration x Chord Familiarity x Number-of-Fingers interaction, *F*(2,60) = 1.03, *p* = 0.36.

In short, the proportions of inaccurate chords appeared relatively high in both practice groups when the right hand was used, unless the right-hand group executed the practiced chords. This confirmed that Right-hand participants had learned to reduce inter-finger interference only in the practiced chords.

### Awareness

At the end of practice, participants possessed a fair amount of awareness of the six chords they had been practicing (Table 2). That is, of the 32 participants 20 correctly wrote down all their 6 chords. Four participants wrote down only 5 chords while 8 tried writing more than 6 chords, up to 11. For those having written more than 6 chords, the number of correct chords was adjusted by subtracting the number of written chords exceeding 6 (e.g., with 8 written chords and 6 correct, the adjusted number of correct chords was 4: 6 correct–2 additional chords). The mean unadjusted correct number of the 1-, 2- and 3-key chords per participant amounted to 1.97, 1.84, 1.94, respectively, of the maximum of 2.

**Table 2:** The numbers of participants with correct chords (of 32 or 64 maximum) as a function of the practice group and the number of keys in each chord, followed by the mean adjusted score

	1-key	2-key	3-key	Adjusted number correct (of 6)
Right-hand	31 (32)	29 (32)	32 (32)	5.4
2-Hand	32 (32)	30 (32)	30 (32)	4.9
Total	63 (64)	59 (64)	62 (64)	5.1

After reproducing their chords, 7 participants indicated to have used 1 strategy to determine their chords, 12 indicated 2 strategies, 8 checked 3 strategies and 4 participants checked 4 strategies. The strategies indicated by the participants were: remembering the letters displayed on the screen (23 of 32 participants; 2-Hand: 12, right handed: 11), remembering the key positions (15: 8, 7), remembering screen locations (16: 5, 11), and reconstruction by pressing keys in one’s mind (12: 7, 5) and on the table top (8: 5, 3). So, participants apparently had some explicit knowledge of verbal and spatial chord knowledge but they also used implicit knowledge to reconstruct their chords. Correlations between individual mean RTs in Block 8 and the adjusted and unadjusted numbers of correct chords written down were far from statistically significant (rs < 0.06). This shows that explicit knowledge was hardly used for executing chords at the end of practice.

A 2 (Practice Group) × 2 (Block: Block 8 vs. Test Block) × 3 (Number if Fingers) × 2 (Repetition) ANOVA on RTs in the last practice block and in the test block condition with the same chords and performing hand as during practice showed no significant effect of performing the awareness test on chord execution (Block main effect, 562 vs. 566 ms, *F*(1,30) = 0.44, *p* = 0.51). Interactions including Block were all far from significant too (ps > 0.23).

Taken together, the awareness test suggested that after practice participants had substantial awareness of the practiced chords and this was not affected by the number of keys in a chord and whether practice had involved 1 or 2 hands. However, 20 of the 32 participants also indicated to have reconstructed the chords by playing them off while answering the questionnaire, and correlations indicated that at the end of practice chord performance was not associated with higher awareness scores, suggesting that awareness did not contribute much to chord execution. Finally, RTs of practiced chords in the test block appeared unaffected by the fact that it had been preceded by the awareness test.

## Discussion

To understand how the brain controls movements of individual fingers when, for example, playing musical instruments and grasping peculiarly shaped objects, the present study tested whether practicing chords involves the development of spatial chord representations indicating which keys are to be pressed, or whether postures are developed of one or even two hands together. Transfer of chord skill to other fingers is predicted by the spatial chord hypothesis but not by the two posture hypotheses. All three hypotheses predict that RT increases with the number of keys in a chord. According to the spatial chord hypothesis this is unaffected by the fingers and hands used. Instead, the posture hypotheses indicate that this RT increase is caused by interference between adjacent fingers and that, consequently, for the same set of chords RT increases less with number of keys in bimanual than unimanual chords. Bimanual chord execution may be slowed by the need to coordinate both hands, like when a more rapidly prepared hand awaits preparation of the slower hand.

### Hand posture learning

The important finding that chord skill did not transfer to the hand configuration of the other practice group (Figs. 3 and 4) refute the spatial chord hypothesis and supports the use of learned hand postures in skilled chord production. The data did not show any indication for time-consuming bimanual coordination because bimanual chords were produced faster than unimanual chords, instead of slower.

As expected, RT and error rate increased as the number of keys went from 1 to 3. The observation that this number-of-fingers effect was greater in the Right-hand than in the 2-Hand practice group (Figs. 1, 4, 5) confirms that it is not due to serially reading out a spatial representation. It is most likely caused by interfinger interference which was indeed generally higher for the Right-hand than the 2-Hand group (with mean CCIs amounting to 3.2 and 2.0, respectively). Robustness of the number-of-fingers effect is confirmed by the re-analysis in Appendix C of the RTs and errors of unimanual chords after 680 practice trials (Seibel 1962). While the present experiment does not allow disambiguating cognitive and biomechanical interference between adjacent fingers, the finding that the number-of-fingers effect did not reduce much in the course of the present 240 practice trials per chord (Fig. 2) is more consistent with a biomechanical than a cognitive cause as learning at the cognitive level could be expected to show a reduction with practice because participants could optimize hand postures by reducing movement differences between individual finger movements.

Still, closer scrutiny of the present data suggested that this number-of-fingers effect may not entirely be explained by interference between fingers because it was observed even in chords with the same CCI. Likewise, the multiple regression analysis of the Seibel (1962) data in Appendix C showed that CCI and number of fingers had independent contributions to RT and error rate. Possible reasons that performance reduced with more keys in same-CCI chords, while serial scanning is refuted, are that faster fingers had to await slower fingers (MacKenzie 1985) and/or that it takes longer to identify the chord-specific stimuli that include more filled placeholders (e.g., Luo and Tian 2022).

As shown in Table 1, chords with a fixed number of fingers but a higher CCI were generally carried out more slowly. However, for two of these chords this CCI effect disappeared with practice while it was robust for two other chords. Participants seem to have been able to overcome enslavement in some chords but not in others, for example, because participants were able to improve independent control of some fingers (Aoyama et al. 2019) and/or were able to optimize some hand postures by minimizing movement differences between adjacent fingers (Van Den Noort et al. 2016). Future research should explore RTs with 4-key chords as in these chords inter-finger interference reduces while the number of fingers increases.

### Bimanual versus unimanual chording

Given that inter-finger interference (and thus CCI) was generally smaller for the 2-Hand than the Right-hand group and that interference between fingers slows chord execution, it lies at hand to attribute the faster execution of bimanual than unimanual chords to the generally lower interference between fingers in bimanual chords. Still, the faster execution of novel bimanual chords in the test phase by the 2-Hand than by the Right-hand participants demonstrates that 2-Hand participants also developed a skill to coordinate the two hands. This bimanual coordination skill can explain that when interference between adjacent fingers was the same in CCI-2 chords, these chords were still executed faster by 2-Hand than by Right-hand participants. This skill may involve the capacity of 2-Hand participants to simultaneously plan postures of both hands while in a bimanual condition Right-hand participants still prepare the two hand postures successively. Future research might examine the possibility that separate hand postures are simultaneously prepared but still independent, by testing whether after bimanual practice the postures of each hand are separately available. Research might also address whether the current bimanual over unimanual performance benefit may have been caused by 2-Hand participants using the two—more independent—index fingers while Right-hand participants also used the more enslaved ring and little fingers.

### Awareness

At the end of practice, participants showed considerable awareness of their chords in that 20 of the 32 participants knew exactly what chords they had been practicing. However, the absence of significant correlations between the awareness test and mean RTs in Block 8 showed that performance did not benefit from explicit chord knowledge. Indeed, the awareness test probably overestimated the immediately available explicit knowledge. That is, when asked, the participants indicated that writing down chords in the awareness test often involved a reconstruction of chords using implicit and perhaps also episodic knowledge and this reconstruction procedure is probably too slow for fastening the execution of chords that are given in response to stimuli. So, the present data suggests, just as with limited chord practice (Hazeltine et al. 2007) and sequential motor skills (Verwey et al. 2016; Verwey et al. 2015), that the skill to execute chords is based on implicit knowledge. This seems in line with chord skill being motoric and not spatial.

### Wrapping up

The data support the hypothesis that (a) the skill to produce chords is based on learned postures of hands rather than on spatial representations. (b) A bimanual coordination skill developed during practice in the 2-Hand participants. This suggests that bimanual practice induces postures for each hand separately that can be simultaneously prepared and then executed. The slower execution of the chords by Right-hand participants can be explained by the higher inter-finger interference when chords were executed unimanually. (c) The insignificant performance-awareness correlations and the cumbersome and most likely slow reconstruction of the learned chords in the awareness test suggest that, despite the fair performance on the awareness test, skilled chord execution was not based on explicit chord knowledge. (d) The lasting RT increase with chords including more keys can be explained by slowed preparation of hand postures that involve more interference between adjacent fingers. (e) Occurrence of this number-of-fingers effect in hand postures with a fixed CCI—having a similar amount of inter-finger interference—suggests that RT is slowed also by other causes like a longer time to identify key-specific stimuli and the need for faster fingers to wait for more slower fingers. (f) The observation that CCI also had an effect in chords with a fixed number of fingers, and that this CCI effect reduced in two chords and not in two other chords, suggests that participants can overcome inter-finger interference in some chords and not in others. These participants may have learned hand postures that were optimized by minimizing movement differences between adjacent fingers, and in some chords they may have been able to increase (biomechanical) independence of adjacent fingers.

## Conclusions

Chord skill is based on the development of hand postures, and not on spatial or explicit chord representations, and practicing with both hands prompted a bimanual coordination skill. Biomechanical and/or neural interference between adjacent fingers seems responsible for the slower execution of chords involving more keys and of chords executed with one hand. Still, chords involving more fingers may have been slowed also by the longer time to identify key-specific stimuli and the need for faster fingers to wait for slower fingers. These conclusions are preliminary because they do not take into account that the Right-hand participants also used the more enslaved ring and little fingers, and that CCI does not reflect that inter-finger interference is much less for the index and little fingers than for the middle and ring fingers.

## Data Availability

The data and EPrime source codes are available on the Open Science Framework (https://osf.io/b382u/?view_only=2201b670e2074bf4ae7f4ea0472c6d6e).

## Appendix A

### Chords executed by each participant

Chords used in the present practice and test phases. Four fingers rested on, and pressed, the C, V, B, and N keys of a QWERTY keyboard.

Note: 1L: 1 left hand finger, 1R: 1 right-hand finger, 2R: 2 right-hand fingers, 1L2R: 1 left-hand finger, 2 Right-hand fingers, etc.

## Appendix C

### Extended analysis of Seibel (1962)

The RTs and errors reported by Seibel (1962) in his Table 1 allowed a post hoc analysis of the effect of CCI on the 31 chords performed with the right hand in the last 85 trials of the 680 practice trials per chord. Correlations between the computed CCIs, and RTs and error percentages were significant, *r*’s (*N* = 31) > 0.37, *p*’s < 0.04. When including just the fingers known to interact substantially with the adjacent fingers, namely the ring and middle fingers, the CCI correlated also with RTs and errors, *r*’s (*N* = 31) > 0.38, *p*’s < 0.04. Instead, this correlation was not significant when only the fingers were included that are known to interact little with their neighbors, namely the thumb, index finger and pinky, *r*’s (*N* = 31) > 0.22, *p*’s > 0.22.

The results showed the number-of-fingers effects in that chords with more fingers were associated with longer RTs, *r*(*N* = 31) = 0.67, *p* < 0.001, and more errors, *r*(*N* = 31) = 0.42, *p* = 0.02. For the ten 3-key chords, RTs and error rate still increased with CCI across all fingers, *r*’s (*N* = 10) > 0.73, *p*’s < 0.02, even when just the ring and middle fingers were involved, *r*’s (*N* = 31) > 0.70, *p*’s < 0.03. These correlations were higher than those with CCI, but were not significant for the ten 2-key chords.

A multiple linear regression was used to predict RT based on CCI and number of fingers. A significant regression equation was found, RT = 4.67 × CCI + 13.6 × Finger Number + 264.4 which yielded an *R*^2^ of 0.82, *F*(2,28) = 29.03, *p* < 0.001. Both CCI and Finger Number were significant predictors of RT (*p*’s < 0.001) but the coefficient was higher for Finger Number than for CCI. Similarly, predicting error percentage on the basis of CCI and number of fingers yielded an *R*^2^ of 0.68 with both independent variables significantly contributing to the prediction of error percentage (*p*’s < 0.002), in Error Percentage = 1.60 × CCI + 2.77 × Finger Number-3.90.

In conclusion, RT increased with number of fingers and, independently, also when there was more interference between the adjacent fingers as indexed by CCI. The CCI effect resulted primarily from interference between the ring and/or middle fingers in 3-key chords, and not in 2-key chords. Furthermore, correlations were higher between the number of fingers and RT and errors than between CCI and RT and errors.

## Footnotes

1 In the nonaging intervals procedure the likelihood that the stimulus is displayed is equal at any moment in time. This results in a exponential probability density and prevents participants from expecting the stimulus at a particular time (Gottsdanker et al. 1986; Nickerson and Burnham 1969).
